# Spatial Response of Ecosystem Service Value to Urbanization in Fragile Vegetation Areas Based on Terrain Gradient

**DOI:** 10.3390/ijerph192215286

**Published:** 2022-11-18

**Authors:** Ji Zhang, Zelin Liu, Yu Shi, Ziying Zou

**Affiliations:** 1 School of Geographic Sciences, Hunan Normal University, Changsha 410081, China; 2 Organization Department of the Shannan Municipal Committee of the CPC, Shannan 856099, China; 3 College of the Life and Environmental Science, Minzu University of China, Beijing 100081, China

**Keywords:** ecosystem service value, terrain gradients, urbanization, vulnerable vegetation areas, Qinghai–Tibet Plateau

## Abstract

The contradiction between urban expansion and ecological protection in fragile vegetation areas has become increasingly prominent with regional development. Revealing the relationship between urbanization and ecosystem services will help to provide solutions to this problem. In order to clarify the impact of urbanization on typical mountain areas with fragile vegetation on the Qinghai Tibet Plateau, we built an ecosystem service value (ESV) evaluation index system. We also evaluated the ESV and its spatial response to the urbanization of Shannan Prefecture in Tibet from 1990 to 2015 based on different terrain gradients (TGs) using vegetation biophysical data obtained from remote sensing platforms. The results show that ESV in Shannan increased first and then declined as the TG increased, reaching a maximum value at the third TG. ESV showed a decreased trend during the study period, with a significant decline at the second and third TGs, which were the main distribution areas of vegetation in Shannan. Through spatial correlation analysis, we observed that urbanization and ESV showed a significant spatial aggregation effect. Among them, the high–low type accounted for the largest proportion in the grid with the agglomeration effect, mainly concentrated at the lower TG in the southern of Shannan, where ESV decreases with the increasing urbanization. We highlight the need for targeted, sustainable development policies to rationally organize the urbanization process in the different-gradient plateau regions with fragile vegetation. These results can provide a reference for applying ESV to vegetation restoration and ecological protection in ecologically fragile mountain areas.

## 1. Introduction

Ecosystem services are the benefits that humans obtain from nature, and their change can reflect the quality of a living environment for human beings [1,2,3]. Since the 1990s, ecosystem services have become an important research topic, and ecosystem service value (ESV) assessment has especially become increasingly popular. Costanza et al. [2] divided global ecosystem services into 17 types, calculated each ecosystem service value (ESV), and proposed a global ecosystem service value equivalent table. ESV has become an important indicator for assessing ecosystem conditions and changes. Quantifying the services provided by the ecosystem into ESV can more clearly and intuitively reflect the operation status of the ecosystem. At present, there are many kinds of assessment methods for ESV, which are generally divided into two categories: indirect analysis and calculation based on land cover data. The direct calculation method is mainly aimed at a specific ecosystem service. With the continuous development and improvement of the calculation method, integrated ecosystem service calculation models, such as InVEST, have been successfully applied in many countries and regions. The direct calculation method can obtain more accurate results, but because the methods and data sources used by different researchers are different, it is difficult to obtain data and make horizontal comparisons of research results. In contrast, the indirect analysis and calculation method based on land cover data has received more attention and application due to its relatively easy access to data and relatively low cost. At present, most of the ESV assessment studies focus on the assessment of the total ESV and its change over time [4,5,6,7,8], mainly focusing on a regional scale or specific ecosystem types.

Approximately 56% of China’s population depends on mountain resources [9]. The terrain is a natural factor that affects the patterns and spatial differentiation of land vegetation status, especially in mountainous areas [10]. In mountainous areas, the terrain (including slope, aspect, etc.) determines the regional temperature, lighting conditions, soil conservation and water conservation capacity; affects the ease of human use; basically determines the utilization mode of the ecosystem; and thus affects the regional ESV. At present, the research on exploring the spatial differentiation characteristics of ESV and the change in spatial difference over time is gradually increasing. However, there are relatively few studies on ESV assessment and changes analysis based on different altitudes and topo-graphic characteristics [11]. Recently, some studies have begun to focus on exploring the impact of topography on ESV from the perspective of terrain gradient. For example, Shi et al. found that the ESV increased with altitude ranging from 600 to 900 m and slopes with inclines ranging from 15° to 25° in Huailai County, Hebei Province, China [12]. Wang et al. found that the ESV in the Heng-duan Mountain region presented obvious spatial heterogeneity across different terrains. Exploring the distribution characteristics of ESVs in mountainous areas based on terrain gradients can effectively promote the construction of regional ecological civilization [13]. Current studies on the impact of topography on ESV mainly concentrate on low-altitude mountain areas [11,14], and there is a lack of research on high-altitude topographical regions.

Many studies have shown that the global ecosystem has degraded, and the supply capacity of ecosystem services has declined [15,16,17]. The change in vegetation has many impacts on the ecosystem, such as changing and affecting the distribution of water resources, reducing soil erosion and loss, etc. Vegetation change is one of the main causes leading to changes in ecosystem services provision [18,19,20]. The vegetation change caused by human activities has a profound impact on the structure, function and change trend of the ecosystem, among which urbanization is one of the important forms of human activities. By changing vegetation coverage, ecosystem structures and ecological processes, urbanization can affect the supplies and distribution of ecosystem services. The difference in ESV can reflect the spatial response of the ecosystem to urbanization. There have been many researches about the impact of urbanization on ESV, most of them were concentrated primarily on metropolitan areas [21,22,23], focusing on the effects of area changes [24,25] and socio-economic changes [26,27]. Some researchers initially revealed that rapid urbanization causes a continuous decline in regional ESV [28,29]. Different from the developed, urbanized areas, the impacts of urbanization on the Qinghai–Tibet Plateau with suffering serious environmental deterioration from both climate change and human interventions, attract less attention [30,31]. There are obvious differences in the distribution of land vegetation patterns and the responses of ESV to urbanization in different terrain conditions [32].

The Qinghai–Tibetan Plateau has the highest average elevation among the major plateau areas worldwide; the high altitude and harsh environmental conditions of which make it result in being the most fragile alpine ecosystem [31]. As a major component of the Qinghai Tibet Plateau, Tibet is not usually regarded as an ideal place for urbanization, but it is a core part of the implementation of the “China Western Development Strategy”. With the acceleration of urbanization driven by the “Promoting Urbanization Strategy”, these cities may face more serious ecological and environmental problems in the coming years [33]. Therefore, it is worthwhile to study the differences in ESV responses to urbanization in Tibet. Take Shannan Prefecture in Tibet as a case study; it is a typical plateau mountainous area with large altitude differences and obvious TGs differences. Moreover, Shannan Prefecture experienced rapid urbanization, and the growth rate of GDP reached 7.9% by 2020. Therefore, Shannan Prefecture is an ideal study site for exploring ESV response to urbanization in Tibet since the increase in human activities driven by urbanization has a significant impact on the ESV [34]. In order to determine the long-term impact of urbanization on ESV in high-altitude areas with fragile vegetation, the main objectives of this study were to (i) describe the topographic differentiation effect of ESV from 1990 to 2015 and (ii) through the spatial response difference of ESV to urbanization on different TGs, reveal the relationship between urban expansion and ecosystem service, which are conducive to improving ecosystem services and provide a reference for balancing ecological protection and economic growth, formulating sustainable development policies in such high altitude mountainous areas with fragile vegetation.

## 2. Methodology

### 2.1. Study Area

**Figure 1:**
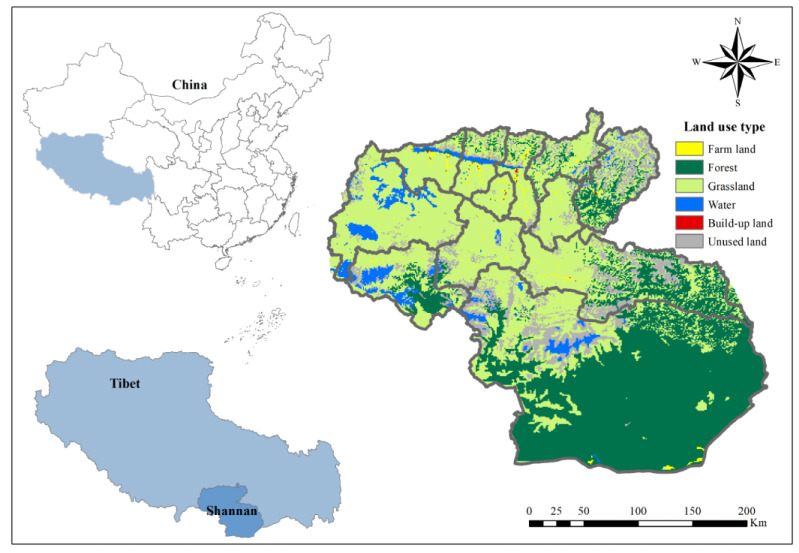
Location and ecosystem pattern of the research area.

Shannan Prefecture is located in the central-southern of Tibet Plateau, in the eastern section of the Himalayas, and in the middle and lower reaches of the Brahmaputra River, 90°14′–94°22′ E, 27°08′–29°47′ N. It borders India and Bhutan, with a whole area of 79,300 square kilometers, occupying one-fifteenth of the whole area of Tibet. Shannan is a typical plateau mountainous city with large elevations and undulations, obvious topo-graphical differences, and diverse ecosystem types. The average altitude is above 3700 m, with the north higher than the south, and the terrain gradient is between 0 and 2.5352. It encompasses most of Tibet’s vegetation types (Figure 1), primarily forest and grassland ecosystems, which provide rich ecosystem services that benefit not only local people but also provide ecological profits for China and even the rest of the world. The main ecosystem services in the study area include food production, material production, climate regulation, water purification, water retention, soil conservation, biodiversity conservation, etc. Compared with other regions on the Tibet Plateau, Shannan is undergoing rapid urbanization.

### 2.2. Data Acquisition

This research applied Landsat TM/ETM remote sensing data from six periods (1990, 1995, 2000, 2005, 2010 and 2015) with a spatial resolution of 1 km. Supported by a large number of classified sample databases constructed from ground survey samples, the data were obtained by using object-oriented multi-scale segmentation and decision tree classification methods (the data source is the Data Center of Resources and Environmental Sciences, Chinese Academy of Sciences, http://www.resdc.cn/ (accessed on 6 July 2021)). On the basis of the Land Application and Land Use Cover Change (LUCC) classification systems, combined with the characteristics of regional land types, the classification system was divided into 6 categories (Figure 1), including farmland, grassland, forest land, water, build-up land and unused land. The analysis was carried out in combination with a 1:250,000 topographic map and thematic map of soil and vegetation in the study area. In addition, the elevation data with 30 m spatial resolution comes from the geospatial data cloud (Chinese Academy of Sciences, http://www.gscloud.cn/ (accessed on 6 July 2021)). Socio-economic data, including population and GDP, are from Tibet statistics yearbooks [35,36,37,38,39,40]. This study calculated the ESV and urbanization degree of the Shannan region based on a 2 km grid. ArcGIS (10.4) (Esri, Redlands, CA, USA) was used for spatial data cutting, grid computing, spatial analysis and statistics, and the analysis software Fragstats (4.2) (U.S. Forest Service, Washington, DC, USA) was used for grid data processing.

### 2.3. Methods

By taking Shannan Prefecture as a case study, we employed the ecosystem service value equivalent conversion method to evaluate ESV and quantitatively analyzed the ecosystem patterns based on different TGs, as well as the variations in ESV based on different TGs. We then conducted a spatial correlation analysis by Moran’s I index to evaluate the responses of ESV to urbanization. The specific approaches are as follows.

#### 2.3.1. Terrain Gradient Classification

As a topographic value for the all-sided description of elevation and slope, the terrain niche indicator shows the topographic condition of a certain point comprehensively, which is generally applied to compare the variations between ecosystem structure and ESV on different topographic conditions. The spot with lower elevation and slope shows a lower terrain niche indicator. The site that has a high elevation but a low slope, or elevation but a high slope, shows a middle-level terrain niche indicator. With the help of the formula, the terrain niche indicator in each grid was calculated on the basis of the outcomes and the Jenks natural break method [41]; the research region was grouped into 5 terrain gradients corresponding to the terrain niche indicator as follows: 0–0.5965; 0.5965~1.0638; 1.0638~1.3819; 1.3819~1.7000; 1.7000~2.5352. The terrain gradient consists of five levels from 1 to 5. The terrain niche indicator formula is [42]:

(1) $$T=\ln\left[\left(\frac{E}{E_{0}}+1\right)\times \left(\frac{S}{S_{0}}+1\right)\right]$$

where T means the terrain niche indicator, E represents the altitude of a point, S refers to the slope of a point, $E_{0}$ means the mean altitude of the whole research area and $S_{0}$ refers to the mean slope of the whole research area.

#### 2.3.2. Evaluation of Ecosystem Service

Costanza et al. divided global ecosystem services into 17 types, calculated the value of each service and proposed a global ecosystem service equivalent table [2]. On the basis of the Costanza theory, Xie Gaodi et al. modified the coefficients according to China’s actual condition and obtained an equivalent table of terrestrial ecosystem service value in China [43]. The ecosystem services include food production, raw materials, water supply, gas regulation, climate regulation, waste treatment, water regulation, erosion control and sediment retention, nutrient cycling, biodiversity conservation and recreation. This research determined the ecosystem service value of Shannan Prefecture by the conversion method of Xie Gaodi et al.; the ecosystem net profit was regarded as the production value provided by the ecosystem, and the net profit of food manufacturing per unit area of farmland ecosystem was understood as the service value of one standard equivalent element. The national average grain output in 2010 was 4974 kg∙hm^−2^. Xie Gaodi et al. determined the economic value of an ecosystem equal in China in 2010 as 3406.50 CNY/hm^−2^. On the basis of the relevant data regarding grain manufacturing in Shannan, the average grain production in the study area is 6811.82 kg∙hm^−2^, and the ecosystem service equal value indicator is 1.3695; therefore, an ecosystem service function equivalent in Shannan is 4665.15 CNY/hm^−2^ [44]. On the basis of the value equivalent and the area of every kind of land, the ecosystem service value of Shannan was obtained. The ecosystem service value evaluation formula is as follows:

(2) $$ESV_{f}=\overset{m}{\underset{f=1}{\sum }}\left(A_{i}\times VC_{fi}\right)$$

(3) $$ESV=\overset{n}{\underset{i=1}{\sum }}\left(A_{i}\times VC_{i}\right)$$

where ESV is the total value of the ecosystem services in the research region; $A_{i}$ is the area of the *i*-th land kind in the research region (hm^2^); $VC_{i}$ refers to the ecosystem service value index of the *i*-th land kind; *n* is the number of land use types; $ESV_{f}$ represents the value of the *f*-th service of the ecosystem; $VC_{fi}$ represents the *f*-th ecosystem service value coefficient of the *i*-th land kind in the research area; and *m* is the number of ecosystem service types.

#### 2.3.3. Urbanization Assessment

Urbanization is the process of systematic evolution and expansion and is mainly manifested as the process of increasing urban population, but also accompanied by a series of social, economic, cultural and natural landscape changes; it is a complex evolutionary process [45]. The measurement of urbanization can be made considering four aspects: population development, economic growth, changes in social life and urban land increase [46]. Population increase and economic growth have laid the basics for urbanization. Changes in lifestyle and an expansion of urban land refer to spatial and social signs of urbanization, respectively [47]. As it is difficult to obtain and spatialize social data, we selected indicators to characterize urbanization in terms of population development, economic growth and land increase. Therefore, population urbanization was quantified by selecting population density (PD); GDP density (GDPD) was used for economic urbanization, and urban land percentage (ULP) was used for land urbanization. Due to the high similarity in the spatial distribution of PD, GDPD and ULP, the three indexes were combined into an integrated indicator—Comprehensive Urbanization Level (CUL). We standardized each indicator to a value between 0 and 1 through scope standardization and then took the average value to obtain the CUL value. The formula for scope standardization is shown below:

(4) $$U_{i,j}^{\prime }=\frac{U_{i,j}-U_{i,min}}{U_{i,max}-U_{i,min}}$$

where $U_{i,j}^{\prime }$ represents the standardized value of $U_{i,j}$, which refers to the original value of the *i*-th urbanization index (such as GDP density, population density, or urban land percentage) in the *j*-th grid. $U_{i,max}$ and $U_{i,min}$ refer to the maximum and minimum values of the *i*-th urbanization indicator across all grids.

#### 2.3.4. Spatial Correlation Exploration between ESV and Urbanization

To explore the space association and agglomeration model between ecosystem service value and urbanization in Shannan, we conducted a spatial correlation analysis. Moran’s *I* index shows the spatial association put forward by Moran (1950), which shows the similarity of the spatially adjoining regional unit value [48]. This research explored the spatial association with global bivariate Moran’s *I* index and local bivariate Moran’s *I* index in Geo Da spatial analysis software. For exploring spatial associations between ESV and the urbanization level of Shannan, global bivariate Moran’s *I* was applied in this research. Additionally, spatial associations within various spatial units were manifested through local bivariate Moran’s *I* [49]. The formulas are as indicated below:

(5) $$I=\frac{N\sum_{i}^{N}\sum_{j\neq i}^{N}W_{ij}Z_{i}Z_{j}}{\left(N-1\right)\sum_{i}^{N}\sum_{j\neq i}^{N}W_{ij}}$$

(6) $$I_{kl}^{i}=Z_{k}^{i}\sum_{j=1}^{N}W_{ij}Z_{l}^{j}$$

(7) $$Z_{k}^{i}=\frac{X_{k}^{i}-{\bar{X}}_{k}}{\sigma_{k}}$$

(8) $$Z_{l}^{j}=\frac{X_{l}^{j}-{\bar{X}}_{l}}{\sigma_{l}}$$

where I is the global bivariate Moran’s *I* for ESV and urbanization; $I_{kl}^{i}$ represents the local bivariate Moran’s *I* for ESV and urbanization extent; N refers to the total number of spatial units; $W_{ij}$ is the spatial weight matrix for the measurement of spatial association between the *i* and *j* spatial units [50]; $Z_{i}$ is the deviation between the property of *i* spatial unit and the average of property; $Z_{j}$ is the deviation between the property of *j* spatial unit and the average of property; $X_{k}^{i}$ refers to the value of property *k* of spatial unit *i*; ${\bar{X}}_{k}$ is the average of property *k*; $\sigma_{k}$ refers to the variance of property *k*; $X_{l}^{j}$ represents the value of property *l* of spatial unit *j*; ${\bar{X}}_{l}$ is the average of property *l*; and $\sigma_{l}$ represents the variance of property *l*.

The values of I/$I_{kl}^{i}$ are from −1 to 1. When the I/$I_{kl}^{i}$ value is positive, it shows a positive spatial correlation between ESV and urbanization. This means that a unit with a high ESV level is followed by units with high urbanization levels. On the contrary, a negative spatial correlation is indicated through a negative I*/*$I_{kl}^{i}$, which indicates that a unit with a high ESV level means low urbanization level units. The high absolute value of I/$I_{kl}^{i}$ reflects a strong spatial correlation. In this study, to assess the statistical significance of bivariate Moran’s *I*, permutation tests (9999 permutations) were applied. In order to ensure that credible results for the spatial association between ESV and urbanization were obtained, a statistically significant value was set at 0.1%.

## 3. Results

### 3.1. Spatial Pattern and Variation in Ecosystem Based on Terrain Gradient

**Figure 2:**
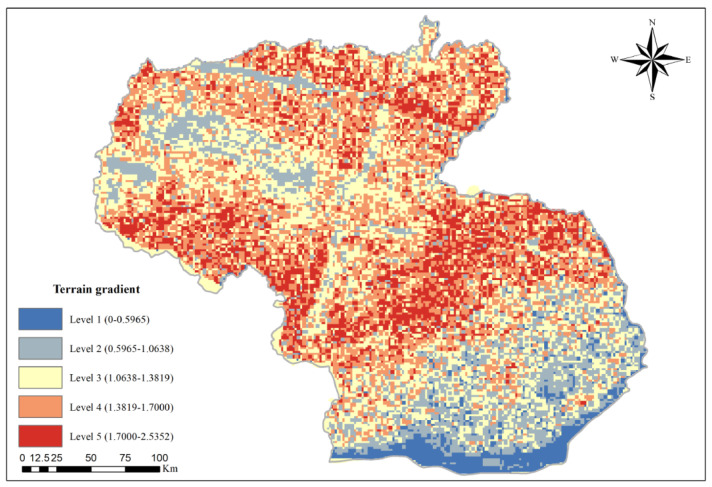
Terrain gradient pattern of Shannan.

The topographic features results based on DEM data show that the topography of Shannan is large undulations and complex. The altitude is high in the north and low in the south. With the lowest altitude being 87 m and the highest altitude being 6777 m, the altitude difference is 6690 m; the western slope is relatively gentle, and the eastern slope is steeper, with a maximum slope of 40.07°. The topographic indicator is high in the middle and low in the north–south direction, with a maximum value of 2.5352 (Figure 2).

**Figure 3:**
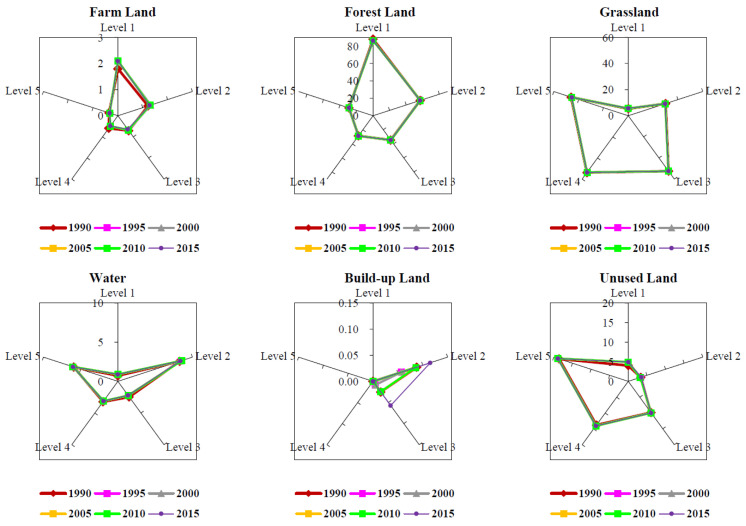
Ratio of ecosystem area in Shannan based on terrain gradients.

The main ecosystem types of Shannan were grassland and forest, accounting for 45.4% and 38.4% of the total area of Shannan in 2015, respectively. Grassland was mainly distributed in the north of Shannan, whereas the forest land was in the south. In addition to grassland and forest land, there were farmland, water, build-up land and unused land in Shannan. Different ecosystem types in Shannan show different changes with the change in TGs (Figure 3). These are as follows: (a) Farmland shows a decreasing trend as the TG increases. As the TG increases, the land is not conducive to farming, and the area of farmland declines. (b) Forest land showed a continuous decreasing trend. Similar to farmland, forest land was distributed at lower TGs with adequate heat and moisture. (c) Grassland initially displayed an increasing trend and then declined. As TG increases, natural enemies and competition decrease, and grassland show an increasing trend. When it increases to the fourth TG, growth is restricted by the environment, such as temperature and water, then the area of grassland begins to decrease. (d) Changes in water and land fluctuate. Water land was mainly distributed at the second and fifth TGs because plateau lakes and reservoirs were mainly distributed at the second TG, and glaciers and snow were mainly distributed at the fifth TG. (e) Built-up land is concentrated at the second and third TGs. These two TGs in Shannan are suitable for human living. (f) Unused land increased as the TG increased. As the TG increases, the environment gradually worsens. This land is unsuitable for development, causing an increase in unused land.

In general, between 1990 and 2015, the area of farmland in Shannan reduced, and the areas of forest land, grassland, water, building-up land and unused land increased (Table 1). Building-up land increased mostly, with an increase of 67%, primarily at the second and third TGs; unused land increased by 184 km^2^, primarily at the first TG; farmland decreased at the higher TGs and increased at the lower TGs. These results show that the population and economy of the prefecture are dense, and urbanization is concentrated at the second and third TGs. Forest land and grassland increased by 313 km^2^ and 293 km^2^, respectively, evenly distributing themselves at TGs of all levels.

### 3.2. Spatial Pattern and Variation in Ecosystem Service Value Based on Terrain Gradient

**Figure 4:**
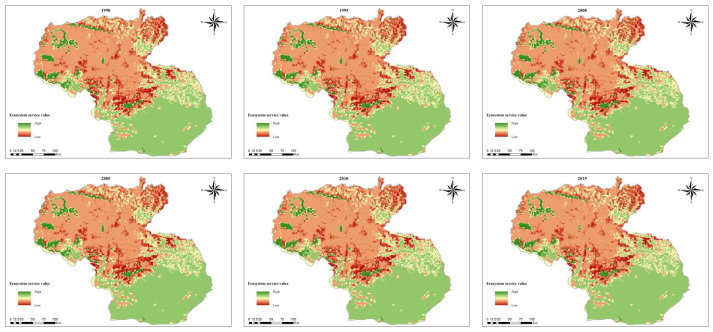
Spatial pattern of ecosystem service value in Shannan.

The accounting results show that the ecosystem service value of Shannan in 2015 was 127.28 billion CNY. From the perspective of overall spatial distribution, the ESV in the southern part of Shannan was higher than that in the northern part (Figure 4). The TG of the southern is generally lower, and in which virgin forests are widely distributed. Specifically, from the perspective of TG, the ESV of Shannan first increased and then decreased as the TG increased, reaching its highest value at the third TG from 1990 to 2015 (Table 2). The total area of forest land and grassland distributed in the third TG was larger than that in other TGs.

The ESV of Shannan displayed a downward trend during the research period, with a decrease of 0.12%. The changes in ESV on the time and TG dimension are shown in Table 2 as follows: ESV at the first TG continues to decline; at the second TG, it shows a decline–rising–declining fluctuant trend; at the third TG, a rising–decreasing fluctuant declining trend is detected; at the fourth TG, a rising–decreasing fluctuant rising trend is shown; and ESV at the fifth TG showed a decline–increasing trend. In general, the ESV at the first, second, third and fifth TGs showed a downward trend, with a significant decline at the second and third TGs, with a decrease of 0.45% and 0.06%, respectively. Combined with the ecosystem changes, this indicates that urbanization at the second and third TGs negatively affects the ESV.

### 3.3. Spatial Correlation between ESV and Urbanization in Shannan

**Figure 5:**
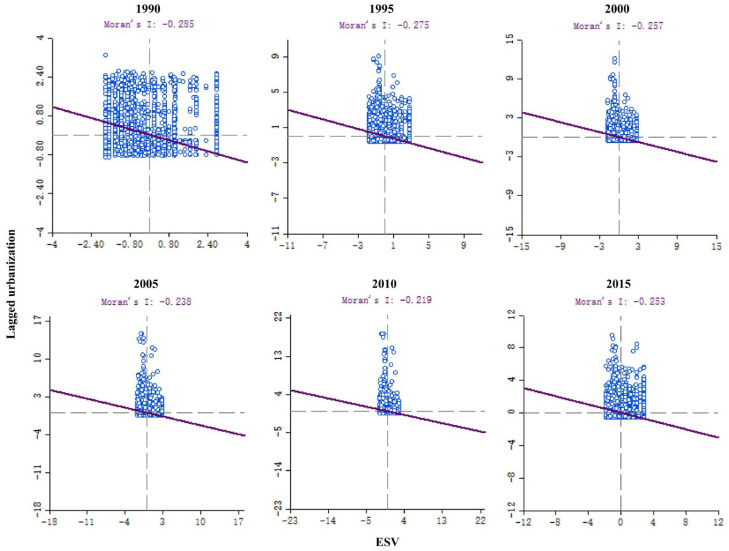
Moran’s I Index of ESV and urbanization in Shannan from 1990 to 2015.

Based on the above analysis of the changes in the ecological pattern of different TGs, land urbanization showed a significant increase, especially in the second and third TGs of the northern part of Shannan. In order to further evaluate the spatial response of ESV to urbanization, we calculated the urbanization index of Shannan and conducted a quantitative analysis of the overall space of the region’s ESV and urbanization. The results show that Moran’s I value in Shannan from 1990 to 2015 were −0.285, −0.275, −0.257, −0.238, −0.219 and −0.253, respectively (Figure 5). Therefore, during the study period, that urbanization negatively affects the ESV. Additionally, in the case of a confidence level of 99%, if the absolute value of Z is higher than 2.58, the null hypothesis is accepted, i.e., the probability that the data are spatially relevant is greater than 99%.

**Figure 6:**
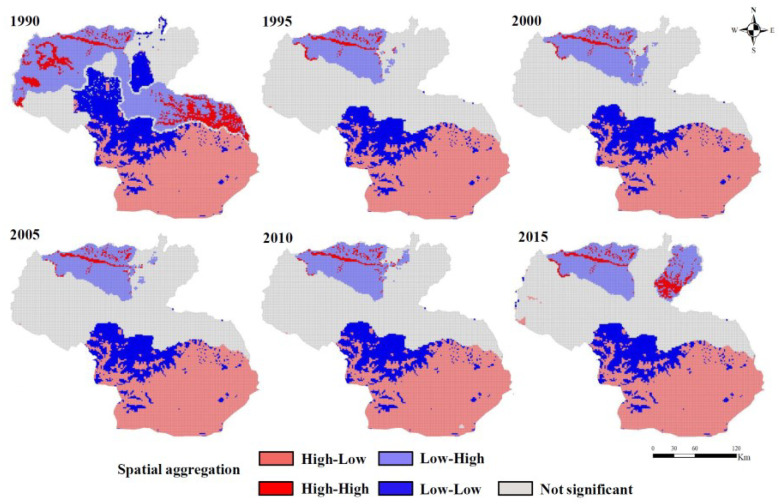
Spatial aggregation of ESV and urbanization in Shannan from 1990 to 2015.

The results of the local spatial clustering effect showed (Figure 6) that the number of grids with spatial clustering roles in Shannan occupied more than 50% of space, with a 95% confidence level; this delimits an increasing trend over 25 years. The number of grids with a high–low clustering effect is relatively large, more than 30%, and these are primarily concentrated in the south of Shannan, which had the lower TGs and mainly covered natural land but not building-up land; the high–low clustering effect is mostly distributed in the second and third TGs in the north, where the ESV decreases as the degree of urbanization increases, resulting in a significant agglomeration effect.

## 4. Discussion

### 4.1. Relationship between TG and ESV

Topography is a significant natural factor that affects the pattern of vegetation and ESV [51,52,53]. Ecosystem types and ESV vary significantly depending on different TGs [54]. Through the investigation focusing on different ecosystem types on all levels of TGs, we found that the distribution of Shannan’s ecosystem types is significantly different in different TGs, mainly forest and grassland, and the areas of these two types increased at all TGs. The area of farmland decreases with the increase in the TGs; it reduced mainly at the third, fourth and fifth TGs, probably due to its higher elevation and slope restricting the development of agriculture [48]. The decreases in forest area are found with TGs increasing, which is different from the previous study concerning low-altitude regions, including Anhui province, Gannan region in Jiangxi province and Huailai County in Hebei province. In such low-altitude areas, urban areas are usually located on the first and second TG, where the degree of human interference is relatively high. In these areas with low TG, the forests will be reduced due to further urbanization, while in areas with high TG, these forests will be protected with less human interference, which indicates that the terrain reduces the external effect on the ecosystem [48,55,56]. Different from the regions outside the Qinghai Tibet Plateau, the distribution of forests in Shannan is mainly affected by hydrothermal conditions. Therefore, with the increase in TGs, the conditions for vegetation growth (lower temperature, water loss, soil depletion, etc.) become worse. Built-up land is mainly distributed at the second and third TGs, which is more suitable for human living with enough water along a river and increases with time. According to previous studies, ecological protection policy such as an artificial afforestation program and a sloping land conversion program in Shannan has achieved certain results [30,57].

The distribution of ESV on different TGs shows obvious differentiation. As the TG increases, the value first increases and then decreases, reaching a maximum at the third TG with the largest forest and grassland area. This is similar to the research results in karst mountainous urban areas [58], but different from those in shallow hilly areas [53]. There is an obvious correlation between ESV and ecosystem types [59,60]: the high ESV was mainly distributed in natural ecosystems with better vegetation, while the low ESV was distributed in urban areas or harsh natural environments.

### 4.2. Response of ESV to Urbanization

During the study period, the ESV of Shannan generally displayed a declining trend, with significant declines at the second and third TGs. This indicates that the urbanization at the second and third TGs negatively influences the ESV [61,62]. Urbanization is an important human factor causing changes in patterns of vegetation and ESV [63,64]. Normally, urbanization affects the ESV by changing ecosystem patterns, negatively influencing ESV [65]; we reached a similar conclusion in Tibet, where the initial urbanization is developing, which is consistent with previous research. During the study period, Shannan’s built-up land (i.e., areas with vulnerable vegetation) increased, primarily at the second and the third TGs, showing that urbanization is concentrated at these two TGs. The development mode of urban expansion is in line with the actual local situation influenced by the climatic and topographic features of the plateau. However, it brought some pressure on the natural ecosystem, especially in the second and third TGs.

Through cluster analysis and quantitative analysis, we found that the number of grids with a high–low clustering effect was relatively large and primarily concentrated in the low TGs in the southern part of Shannan, where the national ecosystem function zones for biodiversity conservation located with a wide distribution of virgin forests [66]. This area has better vegetation and climatic conditions, as well as policy interventions to limit construction activities. A high–low clustering effect was mostly distributed at the second and third TGs in the north, where the ESV decreased with the increase in urbanization. ESV represents a significant spatial response to urbanization. As more and more people congregate in urban areas, more ecosystem services in urban areas are needed for enhanced human well-being. With rapid urbanization, the ecosystem condition at the second and third TGs should receive more attention and be subject to scientific discussion, especially regarding vegetation in urban areas. Therefore, regional development and ecosystem conservation should be approached in prudent and scientific ways.

Due to its location at the national border, the sustainable development of Shannan plays a key role in maintaining the ecological security of the Qinghai–Tibet Plateau and even China. The ecosystem in Shannan is extremely fragile and susceptible to change due to its extreme climate and high altitude [58]. Most of the ecological protection measures for the Qinghai–Tibet Plateau region were formulated based on the overall ecological status [67,68], and protection measures were not formulated according to the ecological status of different TGs. With its high elevation and various TGs, this type of plateau vegetation vulnerable area has fragile ecosystems and harsh natural environments. Therefore, it is suggested to fully balance the relationship between protection and development towards green development based on the actual local situation. In terms of ecological protection, we propose to strengthen vegetation restoration and protection and increase the area and quality of vegetation. In addition, the future land policy of the Shannan region can vigorously focus on realizing the coverage of ecological products (such as ecotourism) based on abundant forest resources under low TG and explore an effective path to transform natural endowments into economic benefits for aboriginal people in limiting agricultural land expansion to maintain better ecosystem service provision [69]. In terms of urban development, policymakers should formulate rational plans to further agglomerate economies and populations in urban areas and encourage planning to increase urban vegetation and provide more ecosystem services to improve ESV. The widely distributed grassland and unused land of TGs 3-5 in central Shannan can be formally planned to provide usable space for the promotion of animal husbandry and afforestation programs [70], which is helpful to establish a “Tibetan-style” development path.

### 4.3. Limitations and Prospects

This study provides some scientific references for nature-anthropogenic coupling in-fluences assessment, e.g., ecosystem pattern and ESV changes based on TGs in mountainous eco-fragile areas. The method of analysis of the spatial correlation between urbanization and ESV is also an important contribution to research and policy-making. However, some inevitable limitations in this study still exist. The evaluation of ESV was assessed mainly by RS data and statistic data; more field survey data should be collected for assessment. We employed the table of equivalent factors of ESV in China, and there may be some deviations. Due to the spatial heterogeneity of ESVs, there are differences in ESVs in different regions, even within the same ecosystem. In addition, this study assessed ESV based on changes in ecosystem types. However, occasionally the ecosystem type does not change, but its quality has a relatively large change, which requires further in-depth research in the future. The analysis of the relationship between ESV and urbanization should include a more scientific process, for example, ecosystem services provided by urban green [71]. Drove by the “China Western Development Strategy” and “Promoting Urbanization Strategy” [72], the impacts of urbanization on Tibet’s ecosystem will remain for a long time. In future research, we will conduct a field survey and interview to obtain multiple data to improve the assessment of ESV and the impact of urbanization on livelihood and conduct an analysis of scenarios considering the predictions of ecosystem patterns and ESV [73]. In addition, due to different DEM interpolation methods, the extraction of digital terrain factors is uncertain, which is one of the limitations of this study.

## 5. Conclusions

ESV varies greatly, both temporally and spatially, affected by the change in ecosystem type and pattern. In this study, we investigated the relationship between ESV and urbanization in the fragile vegetation plateau area. We found that topography is one of the important factors that affect the ecosystem and its ESV in such areas. The topographic gradient effect of ESV shows that with the increase in TG, ESV first rises and then decreases. ESV decreased from 1990 to 2015, especially in the second and third TG. Our results are consistent with other research results but also reveal the distribution differences between Tibet and low-altitude areas. Through quantitative spatial analysis, we found that the relationship between urbanization and ESV shows an obvious spatial correlation. The clustering effect shows that the areas with high–low clustering of ESV and urbanization are mainly distributed in virgin forest areas with lower TGs. Important ecosystems and human urbanization in Shannan are both distributed in regions with lower TGs, where the ESV showed a downward trend. This serves as a warning for future urban development in vulnerable plateau vegetation areas such as Shannan. The relationship between protection and development should be well coordinated, and the space layout of the ecosystem should be planned rationally. Our research results could provide a reference for how to coordinate regional development, vegetation restoration and ecological protection in vulnerable plateau vegetation areas.

## Figures and Tables

**Table 1:** Variation in ecosystem based on terrain gradient from 1990 to 2015 (%).

TG	Farm Land	Forest Land	Grassland	Water	Build-Up Land	Unused Land
Level 1	20.0	0.9	7.6	28.6	-	25.3
Level 2	7.2	1.9	0.5	3.0	33.3	6.1
Level 3	−7.1	2.5	1.9	−8.1	133.3	3.1
Level 4	−15.5	2.4	1.8	−1.0	-	4.0
Level 5	−4.3	2.0	1.2	3.9	-	3.9

**Table 2:** Change in ESV in Shannan based on TG from 1990 to 2015 (CNY/hm^2^).

TG	1990	1995	2000	2005	2010	2015	Change
Level 1	1140.66	1139.97	1139.97	1139.97	1139.97	1139.97	−0.70
Level 2	3621.15	3616.99	3617.26	3620.84	3620.84	3604.98	−16.17
Level 3	4629.06	4629.10	4629.19	4629.02	4629.02	4626.35	−2.72
Level 4	4299.65	4301.09	4301.42	4301.38	4301.38	4300.53	0.88
Level 5	2423.31	2423.00	2423.00	2423.00	2423.00	2423.19	−0.12

## Data Availability

Not applicable.
